# The Descriptive Features of Primary Spontaneous Pneumothorax Patients in King Abdulaziz Medical City, Riyadh, Saudi Arabia

**DOI:** 10.7759/cureus.40493

**Published:** 2023-06-16

**Authors:** Sultan K Alruwaili, Abdulaziz A Alquraishi, Omar K Binsaddik, Nawaf S Alanazi, Abdulaziz Bin Marshed, Ali M Albargawi, Rakan Mounla Ali, Hassan Arishi

**Affiliations:** 1 General Surgery, National Guard Health Affairs, Riyadh, SAU; 2 General Practice, King Saud Bin Abdulaziz University for Health Sciences, Riyadh, SAU; 3 Pediatric Surgery, Maternity & Children Hospital, Riyadh, SAU; 4 Surgery, King Abdulaziz Medical City, Riyadh, SAU; 5 Surgery, King Saud Bin Abdulaziz University for Health Sciences, Riyadh, SAU; 6 Thoracic Surgery, King Abdulaziz Medical City, Riyadh, SAU

**Keywords:** emergency medical service, spontaneous pneumothorax, general surgery, thoracic sugery, pneumothorax, primary spontaneous pneumothorax

## Abstract

Background

Primary spontaneous pneumothorax (PSP) is a fairly prevalent disorder in emergency medicine. PSP most frequently affects tall, thin male smokers and is most prevalent during adolescence. Published literature contains a wide range of Primary Spontaneous Pneumothorax (PSP) recurrence rates, but there is limited information on the variables affecting recurrence.

Objective

To identify the descriptive features of PSP in King Abdulaziz Medical City, Riyadh, Saudi Arabia.

Methods

This retrospective cross-sectional study was conducted in Surgery King Abdulaziz Medical City, Riyadh, Saudi Arabia. Including all PSP patients from 2016-2021, excluding pediatric and geriatric patients. Participants were selected using a simple random sampling technique, and data were collected from hospital records. Data analysis was conducted by using SPSS.

Results

In this study, we included a total of 131 participants. Most were males (93.1%), and most were aged between 21-30 years. Our findings showed that most PSP events occurred in winter (28.6%). Followed by fall (25.7%), summer (25.0%), and spring (20.7%). Concerning the smoking status of our respondents, our results revealed that most of them were active smokers (72.5%). Left-PSP was the most commonly reported type of PSP (43.5 %), followed by right-PSP (38.9%), non-simultaneous bilateral PSP (14.5%), and bilateral simultaneous PSP (3.1%). Moreover, we found that the recurrence rate of PSP was 42%. Regarding the management of PSP, almost half of the respondents were managed initially by Chest tube. The most frequently used surgical option was VATS- Bullectomy with Abrasion Pleurodesis. Finally, the recurrence rate of PSP was 42% among the patients. The percentage of patients with one recurrence only was 65.5% among the patients with recurrent PSP, second recurrence at 29.1%. Third, Fourth, and Fifth had the same recurrence percentage of 1.8%, and these percentages came to be statistically significant. (P value < 0.001)

Conclusion

Our study concluded that PSP was more prevalent in tall, thin, young male smokers. Almost half of the respondents suffered from at least one recurrence attack of PSP. The majority of the patients with recurrences experienced one recurrence only, and the second recurrence was estimated to be almost one-third. There is no significant association between the occurrence and seasons of the attack at a time. Most of the participants were managed initially by a chest tube. The most frequently used surgical option was Video Assisted Thoracoscopic Surgery (VATS) with abrasion pleurodesis.

## Introduction

Pneumothorax occurs when air enters the chest cavity, more specifically, between the parietal and visceral pleural cavities. This condition develops when injured tissue forms a one-way valve, allowing air to enter the pleural space and preventing the air from escaping naturally [1]. Pneumothorax is classified into two types: traumatic and spontaneous pneumothorax. Traumatic pneumothorax occurs after penetrating or blunt trauma to the chest, leading to air entering the pleural space through the chest wall directly into the pleural space. Spontaneous pneumothorax, on the other hand, is non-traumatic and develops with no obvious preceding factor [2]. Spontaneous pneumothorax is further divided into primary spontaneous pneumothorax (PSP) and secondary spontaneous pneumothorax (SSP). This paper focuses on PSP. The annual incidence of PSP ranges from 15.5 to 22.7 per 100,000, with a female/male ratio that ranges from 1:3.3 to 1:5 [3].

In SSP, patients usually present with a preexisting lung pathology, such as Chronic Obstructive Pulmonary Disease (COPD), associated with a higher morbidity and mortality rate than PSP. However, in PSP, patients present no underlying lung disease [4].

PSP often occurs at rest, and patients diagnosed with PSP are usually tall, thin male smokers who tend to be between 10 and 30 years old. Atmospheric pressure changes have also been shown to increase the risk of developing PSP and exposure to loud music [4]. Many researchers believe that PSP is always caused by the spontaneous rupture of blebs bullae in the visceral pleura [5]. PSP patients could present as asymptomatic or, in some cases, could present with multiple symptoms, such as the acute onset of pleuritic chest pain accompanied by dyspnea, tachypnea, tachycardia, tracheal deviation, or ipsilateral diminished chest expansion and breath sounds [6].

History and physical examination are extremely important in reaching a diagnosis, confirmed by imaging of the posteroanterior and lateral chest for any abnormal air in the pleural space [7,8]. Although it is relatively rare, tension pneumothorax should be considered a life-threatening complication requiring decompression; other complications include pneumomediastinum, pneumoperitoneum, and subcutaneous emphysema, commonly associated with major trauma [8].

The PSP recurrence rate varies widely due to the limited data describing the influential factors. "A systematic review and meta-analysis that describe the recurrence rates of 3,607 studies have shown overall recurrence rates of 29.0% (95% CI 20.9-37%) and 32.1% (95% CI 27.37.2%), respectively. Female gender was associated with increased recurrence, while smoking cessation was associated with a four-fold decrease in risk" [9], indicating a recurrence rate of 32%, with the greatest risk in the first year [9].

The management of PSP ranges from observation to surgical management, depending on its severity. It starts with conservative management in cases of small PSP with no significant breathlessness. Severe breathlessness in a patient with a small PSP may indicate tension pneumothorax. Chest drain insertion and admission are required in tension and bilateral pneumothorax; moreover, high-flow oxygen should be given to hospitalized patients [7]. "According to Costumbrado J, larger primary spontaneous pneumothorax can be further managed with video-assisted thoracoscopy surgery (VATS) or thoracotomy to perform bullectomy, pleurectomy, and mechanical pleurodesis (i.e., dry gauze abrasion). VATS is less invasive than thoracotomy and has been shown to be an effective measure in treating and preventing spontaneous pneumothorax recurrence" [2].

To date, the exact pathogenesis of PSP is unknown. However, several studies have shown that smoking, gender, weather, decreased atmospheric pressure, and occupation might play a major role in its occurrence [10]. Due to the lack of such data in Saudi Arabia, we aim to identify the descriptive features of PSP in that country, such as demographics, season of onset, smoking status, and recurrence rate, using data collected at King Abdulaziz Medical City (KAMC) in Riyadh, Saudi Arabia.

## Materials and methods

The research team collected and analyzed data and records of PSP patients in KAMC's Department of Surgery. The team used a retrospective cross-sectional study design using a chart review to conduct this research.

Study area/ setting

This research was conducted in the Department of Surgery in KAMC, Riyadh, Saudi Arabia, a tertiary academic hospital that has eleven divisions under the Department of Surgery. KAMC's Thoracic Surgery Division aims to provide the finest health services to all PSP patients.

Study subjects

This research focuses on recognizing patterns and descriptive characteristics associated with PSP in all male and female PSP patients ages 14-65 who were treated at KAMC from 2016-2021, excluding pediatric and geriatric patients.

Sample size

The sample size was estimated using the Raosoft, Inc. sample size calculator, which has a 5% margin of error. The confidence level is 95%, and the response distribution is 50%. The sample size is 131. This study will include PSP patients from 2016-2021, excluding pediatric and geriatric patients.

Sampling technique

A simple random sampling technique or non-probability consecutive sampling was used depending on the number of patients and the data available.

Data collection methods, instruments used, and measurements

Data collection, which was done by the research team, will mainly consist of observing and analyzing patients' data records (e.g., age, gender, smoking history, type of management, season of the attack, occupation, and other health issues, and their correlation to PSP) from the BESTcare system in KAMC.

Data management and analysis plan

IBM Statistical Package for the Social Sciences (SPSS) Statistics 20.0 was used for data entry and analysis. Categorical data were presented in frequencies "n," percentages, and bar charts. Examples of this data include gender, age, and habitual factors. For inferential statistics, the Chi-square test was used[AL9] for the research to link two variables (e.g., PSP and gender, a specific season, or family history). The Chi-square test assessed the relationships between PSP and other characteristics. The test was considered significant if the p-value was less than 0.05.

Ethical consideration

The data collected from KAMC's BESTCare system of patients with PSP was only available to those who participated in this research, which limited the records' access to the research team members only. Also, confidentiality was ensured, and the collected data was kept safe.

## Results

In this study, a total of 131 participants were included. Most of the patients were males (93.1%), and most participants were between the age range of 21 to 30 years old. Results showed that most PSP attacks occurred in winter (28.6%), followed by fall (25.7%), summer (25.0%), and spring (20.7%). Regarding smoking status, findings revealed that most participants were mostly active smokers (72.5%). Left-PSP was the most commonly reported type of PSP (43.5 %), followed by right-PSP (38.9%), non-simultaneous bilateral PSP (14.5%), and bilateral simultaneous PSP (3.1%). Moreover, the PSP recurrence rate was found to be 42%. Regarding management, almost half of the respondents were managed initially by a chest tube. The most frequently used surgical option was VATS bullectomy with abrasion pleurodesis. Finally, the PSP recurrence rate was 42%, with 65.5% of patients having one recurrence, 29.1% having a second recurrence, and 1.8% having a third, fourth, and fifth recurrence, respectively. These percentages are statistically significant with a p-value < 0.001.

Characteristics of the respondents

A total of 131 respondents were involved in this study. Most were males (93.1%), and almost half were aged between 21 to 30 years old. Those between the ages of 14 to 20 constituted almost one-third of the studied sample. Body mass index was assessed, and most patients were underweight or healthy, constituting 47.3% and 48.1%, respectively (Table 1).

**Table 1:** Socio-demographic data of the respondents (n=131)

Variable	Categories	Frequency	Percent
Gender	Male	122	93.1%
	Female	9	6.9%
Age (years)	14 – 20	42	32.1%
	21 – 30	66	50.4%
	31- 40	14	10.7%
	41 – 50	5	3.8%
	More than 50	4	3.1%
Nationality	Saudi	126	96.2%
	Non-Saudi	5	3.8%
Body Mass Index (BMI)	Underweight	62	47.3%
	Healthy weight	63	48.1%
	Overweight	4	3.1%
	Obese	2	1.5%

Seasons of the attack of PSP and smoking status of participants

Throughout the yearly seasons, the analysis listed in (Table 2) revealed that there were no significant differences, with winter being the relatively most common reported season (28.6%) and spring as the least frequent (20.7%). Most sampled patients were active smokers (72.5%), with 71.3% using conventional cigarettes, 18.3% using e-cigarettes, and 10.4% using shisha at an average number of 1.7 ± 0.83 (range 0.5-4) packs per day and an average smoking duration of 6.9 ± 6.76 years (range 0-38).

**Table 2:** Seasons of the attack and smoking status of participants

Variable		N	%
Season of the attack	Fall season	36	25.7%
	Winter	40	28.6%
	Summer	35	25.0%
	Spring	29	20.7%
Smoking	Active smoker	95	72.5%
	Passive smoker	20	15.3%
	Non-smoker	16	12.2%
Type (n=115)	cigarette	82	71.3%
	E-cigarette	21	18.3%
	shisha	12	10.4%
Smoking Quantity	1.7 ± 0.83 pack per day (Range 0.5 to 4)		
Smoking Duration	6.9 ± 6.76 years (Range 0 to 38)		

Types, recurrence rate, and management of primary spontaneous pneumothorax

Relating PSP types, recurrence rate, and type of management, (Table 3) revealed that the highest proportion of patients was diagnosed with left PSP (43.5 %), followed by right PSP (38.9%), non-simultaneous bilateral PSP (14.5%), and lastly bilateral-simultaneous PSP (3.1%). Moreover, we noticed that the recurrence rate was 42%. An initial management survey indicates that 57.9% of patients were managed by a chest tube, followed by surgery (23.7%), and finally, conservative management (18.4%). VATS bullectomy was the most frequent surgical option (99%), while thoracotomy bullectomy was performed in only 1% of cases. In addition to these two types of surgery, abrasion pleurodesis was performed in 84.5% of the cases, whereas talc pleurodesis was performed in 4.1%. A pleurectomy was performed in 24.7% of cases.

**Table 3:** Types, recurrence rate, and management of PSP. PSP: Primary spontaneous pneumothorax; VATS: Video Assisted Thoracoscopic Surgery

Variable		N	%
Type of PSP	Non-simultaneous bilateral PSP	19	14.5 %
	Bilateral- simultaneous	4	3.1 %
	Right-PSP	51	38.9 %
	Left- PSP	57	43.5 %
Recurrence rate of PSP		55	42%
1st recurrence		36	65.5%
2nd recurrence		16	29.1%
3rd recurrence		1	1.8%
4th recurrence		1	1.8%
5^th^ recurrence		1	1.8%
Initial management	Chest tube	107	57.9%
	Surgery	44	23.7%
	Conservative	34	18.4%
Type of surgery	VATS Bullectomy	96	99%
	Thoracotomy Bullectomy	1	1%
+/- Abrasion Pleurodesis +/- Talc Pleurodesis +/- Pleurectomy	Abrasion pleurodesis	82	84.5%
	Talc pleurodesis	4	4.1%
	Pleurectomy	24	24.7%

Association between type of PSP and other factors

The association between the type of PSP and other factors indicates that patients with bilateral-simultaneous PSP had at least one recurrence, and this association came to be statistically significant (p-value < 0.001). All other factors such as gender, age, nationality, BMI, smoking status, smoking quantity, and smoking duration had no significant association with PSP type, but gender and passive smoking showed a relatively higher degree of association (p-values = 0.226 and 0.252, respectively), as demonstrated in (Table 4).

**Table 4:** Association between type of PSP and other factors PSP: Primary spontaneous pneumothorax

Variable	Non-simultance bilateral PSP	bilateral simultance	LT-PSP	RT-PSP	P value
n (%)					
Gender					
Male	19 (15.6)	4 (3.3)	54 (44.3)	45 (36.9)	0.226
Female	0 (0)	0 (0)	3 (33.3)	6 (66.7)	
Age (years)					
14 – 20	6 (14.3)	1 (2.4)	20 (47.6)	15 (35.7)	0.41
21 – 30	8 (12.1)	2 (3)	28 (42.4)	28 (42.4)	
31- 40	3 (21.4)	1 (7.1)	7 (50)	3 (21.4)	
41 – 50	2 (40)	0 (0)	2 (40)	1 (20)	
More than 50	0 (0)	0 (0)	0 (0)	4 (100)	
Nationality					
Saudi	19 (15.1)	4 (3.2)	55 (43.7)	48 (38.1)	0.649
Non-Saudi	0 (0)	0 (0)	2 (40)	3 (60)	
Body Mass Index (BMI)					
Underweight	12 (19.4)	3 (4.8)	22 (35.5)	25 (40.3)	0.64
Healthy weight	7 (11.1)	1 (1.6)	31 (49.2)	24 (38.1)	
Overweight	0 (0)	0 (0)	3 (75)	1 (25)	
Obese	0 (0)	0 (0)	1 (50)	1 (50)	
Active smoker					
Yes	16 (16.8)	3 (3.2)	42 (44.2)	34 (35.8)	0.555
No	3 (8.3)	1 (2.8)	15 (41.7)	17 (47.2)	
Passive smoker					
Yes	1 (5)	1 (5)	7 (35)	11 (55)	0.252
No	17 (16.3)	3 (2.9)	48 (46.2)	36 (34.6)	
	Mean ± SD				
Smoking Quantity (Pack/day)	1.68 ± 0.89	1.25 ± 0.50	1.53 ± 1.02	1.30 ± 0.92	0.41
Smoking Duration (years)	6.84 ± 7.18	7.00 ± 6.00	4.89 ± 5.20	6.88 ± 8.00	0.426

## Discussion

The spontaneous presence of air in the pleural spaces of patients with no clinically apparent underlying lung disease is known as PSP [11]. Many researchers believe that PSP is always caused by the spontaneous rupture of a sub-pleural bleb or bulla [5]. Data and records of 131 PSP patients seen under the Division of Thoracic Surgery at KAMC during the period 2016 to 2021 were analyzed, and data such as age, gender, smoking history, a season of the attack, type of management, and other health factors were correlated to PSP.

The results demonstrated that most respondents were males (93.1%) between 21 and 30 years of age, with 50% of respondents having a normal BMI. This finding is in close agreement with a previous study in Saudi Arabia, indicating that most PSP patients are males (98.7%) with a mean age of 24 ± 6 years and a mean BMI of 19.2 ± 3.8 kg/m2 [12]. Another study showed an annual PSP incidence among males of 7.4 per 100,000 and an incidence among females of 1.2 per 100,000 [13]. Regarding weight, a published study found that patients with low BMI are significantly more likely to suffer PSP [14]. Hence, according to this study and previously published research, PSP is primarily a disease of young, normal-BMI males.

In terms of the seasons, this present study revealed that PSP patients were admitted in relatively equal proportions throughout the different seasons, with winter as the most commonly reported season for the onset of PSP (28.6%). A previous study in Saudi Arabia reported that the onset of PSP was more common in spring and summer [15]. An earlier study in Italy found that PSP was significantly more likely to occur on warm, windy days [16]. Other authors did not find significant differences in admissions' seasonal and monthly distribution [17-19]. Hence, this study and the literature demonstrate that the season of incidence is not a strong factor in PSP occurrences. 

A link between cigarette smoking and developing pneumothorax remains controversial, given that non-smokers are also at risk [20]. In one study, smoking increased the rate of developing PSP by 22-fold in males [21]. Previous findings in Saudi Arabia revealed that 62% of PSP patients were smokers [12], but to the contrary, another study reported that smoking was documented in only 16% of patients [22]. It is worth noting that these two studies used a small number of sample patients. This work supports the perception that smoking is a strong factor, finding that most patients studied were active smokers (72.5%) using conventional cigarettes, e-cigarettes, and shisha. 

Furthermore, the results showed that the highest proportion of patients were diagnosed with left PSP (43.5%), followed by right PSP (38.9%). This contradicts a previous study conducted with 25 patients in Saudi Arabia, which found that the ratio of right-to-left pneumothorax was 6:1, with only one bilateral pneumothorax documented [22]. Moreover, PSP recurrence was observed in 42% of sampled patients, with at least one recurrence for patients diagnosed with bilateral-simultaneous PSP. A previous systemic review concluded that the overall recurrence rate of PSP was 29% in the studied sample [9]. Another study conducted in the United States showed a higher recurrence incidence of 54.2%, twice as common in males than in females [23].

Surgically, almost half of the patients in this study were initially managed by chest tube insertion and VATS bullectomy, with abrasion pleurodesis being the most frequent surgical option. A previously published study in Saudi Arabia found that 90.9% of the patients were treated surgically [15]. 

Finally, it is important to indicate some of the limitations of this study, mainly that the sample size should be larger for more accurate and generalized findings. Additionally, the study was observational in nature, and that limited our ability to establish a causal relationship. Last, the recurrence rate is not well-estimated because we only included patients admitted to KAMC hospital.

## Conclusions

Our study concluded that PSP is more prevalent in male smokers who are tall, thin, and young. Almost half of our respondents were aged between 21 to 30 years old, and about one-third were between 14 to 20 years old. Most of the patients were smokers. Almost half experienced at least one recurrence of PSP, whether ipsilateral or contralateral. More than half of the patients who had recurrence experienced only one recurrence. The patients who experienced a second recurrence were almost one-third of the total patients who had a recurrence. Third, fourth, and fifth recurrences each had the same recurrence percentage.
